# Hypoxia-inducible factor 1 alpha is a poor prognostic factor and potential therapeutic target in malignant peripheral nerve sheath tumor

**DOI:** 10.1371/journal.pone.0178064

**Published:** 2017-05-30

**Authors:** Suguru Fukushima, Makoto Endo, Yoshihiro Matsumoto, Jun-ichi Fukushi, Tomoya Matsunobu, Ken-ichi Kawaguchi, Nokitaka Setsu, Keiichiro IIda, Nobuhiko Yokoyama, Makoto Nakagawa, Kenichiro Yahiro, Yoshinao Oda, Yukihide Iwamoto, Yasuharu Nakashima

**Affiliations:** 1 Department of Orthopaedic Surgery, Graduate School of Medical Sciences, Kyushu University, Fukuoka, Japan; 2 Division of Orthopaedic Surgery, National Cancer Center Hospital, Tokyo, Japan; 3 Department of Orthopaedic Surgery, Kyushu Rosai Hospital, Kitakyushu, Japan; 4 Department of Anatomic Pathology, Pathological Sciences, Graduate School of Medical Sciences, Kyushu University, Fukuoka, Japan; 5 Kyushu Rosai Hospital, Kitakyushu, Japan; University of Pécs Medical School, HUNGARY

## Abstract

**Background:**

Malignant peripheral nerve sheath tumor (MPNST) is a rare soft tissue sarcoma with poor prognosis. Hypoxia-inducible factor 1 (HIF-1) plays a crucial role in the cellular response to hypoxia and regulates the expression of multiple genes involved in tumor progression in various cancers. However, the importance of the expression of HIF-1α in MPNSTs is unclear.

**Methods:**

The expression of HIF-1α was examined immunohistochemically in 82 MPNST specimens. Cell culture assays of human MPNST cells under normoxic and hypoxic conditions were used to evaluate the impact of anti-HIF-1α–specific siRNA inhibition on cell survival. A screening kit was employed to identify small molecules that inhibited HIF-1α.

**Results:**

The nuclear expression of HIF-1α was positive in 75.6% of MPNST samples (62/82 cases). Positivity for HIF-1α was a significant poor prognostic factor both in univariate (*P* = 0.048) and multivariate (*P* ≤ 0.0001) analyses. HIF-1α knockdown abrogated MPNST cell growth, inducing apoptosis. Finally, chetomin, an inhibitor of HIF-1α, effectively inhibited the growth of MPNST cells and induced their apoptosis.

**Conclusion:**

Inhibition of HIF-1α signaling is a potential treatment option for MPNSTs.

## Introduction

Malignant peripheral nerve sheath tumor (MPNST) is one of several highly malignant soft tissue sarcomas (STSs), with an annual incidence of about 5 per million [1]. Approximately half of MPNSTs are associated with neurofibromatosis type 1 (NF 1), while the other half are sporadic. Surgical resection is the curative treatment of choice for MPNSTs; however, the rates of both local recurrence and distant metastasis are approximately 50%, and the 5-year survival rate is as low as 40% [2–4]. The clinical benefits of chemotherapy for MPNSTs are still debated [5]. As in cases of other histological types of STS, the combination of doxorubicin and ifosfamide is recommended for MPNSTs; however, the response rate is unsatisfactory (21%) [6]. In addition, no new drugs effective against MPNSTs have been developed thus far [7]. Therefore, identifying new therapeutic targets and effective novel agents against MPNSTs is essential to improve the survival of patients with this condition.

Hypoxia-inducible factor (HIF) plays a dominant role in the cellular response to hypoxia. HIF is a heterodimer of an oxygen-labile α subunit and a constitutively expressed β subunit [8]. Under normoxia, HIF-α protein undergoes rapid proteasomal degradation after ubiquitination with E3 ubiquitin ligase-containing von Hippel-Lindau protein. Conversely, under hypoxia the degradation process is attenuated and HIF-α localization shifts from the cytoplasm to the nucleus, thereby regulating energy metabolism, tumor cell invasion and migration, cell proliferation and survival, and the expression of multiple genes involved in angiogenesis, including vascular endothelial growth factor (VEGF) [9–13]. Overall, the nuclear expression of HIF contributes to tumor progression. The importance of hypoxia-independent nuclear HIF-1α expression has also been reported in various malignant tumors [14, 15]. Moreover, a number of studies have confirmed that the nuclear expression of HIF-1α is correlated with poor prognosis in various cancers, including cervical cancer [16], endometrial carcinoma [17], and lung cancer [18]. Furthermore, the importance of HIF-2α has also been verified in recent years [19, 20]. In contrast, previous studies have been inconsistent regarding the prognostic role of nuclear HIF expression in STSs [21]. However, expression of VEGF, downstream of HIF-1α, is reported to be up-regulated in MPNSTs [22], suggesting that MPNST is one of the histologic types of tumors in which HIF-1α is responsible for tumor progression.

Against this background, in this study we conducted a large-scale clinicopathologic and prognostic analysis of the nuclear expression of HIF-1α in 82 MPNST clinical specimens. In addition, HIF-2α staining was also performed in 69 available specimens. We then tested the cell-autonomous roles of HIF-1α in MPNST cell lines. We further attempted to identify new target molecules for inhibiting HIF-1α signaling in MPNSTs through cell-based comprehensive screening using a chemical library containing more than 300 compounds.

## Materials and methods

### Patients and tumors

Eighty-five paraffin-embedded primary MPNST specimens from 85 patients collected between 1964 and 2011 were retrieved from the soft tissue tumor registry at the Department of Anatomic Pathology, Pathologic Sciences, Kyushu University (Fukuoka, Japan). All cases in this report were included in previous studies [23, 24]. The diagnosis of MPNST was revised according to the 2013 edition of the World Health Organization classification, and one case was excluded. Clinical details and follow-up information were obtained by reviewing medical charts. Follow-up information was available in 82 of 85 cases. The clinicopathological characteristics are summarized in Table 1. This survey was conducted in accordance with the principles of the Declaration of Helsinki. The study was also approved by the Ethics Committee of Kyushu University (No. 21–137) and conducted according to the Ethical Guidelines for Epidemiological Research enacted by the Japanese Government.

**Table 1:** Association between clinicopathological variables and immunohistochemical detection of HIF-1α.

Variables	Number of patients	HIF-1α		*P*
		Positive	Negative	
**Clinical variables (*n* = 82)**				
**NF1**				
present	32	22	10	0.296
absent	50	40	10	
**Age**				
≥50	37	30	7	0.317
<50	45	32	13	
**Sex**				
male	43	36	7	0.121
female	39	26	13	
**Location**				
trunk	47	37	10	0.604
extremities	35	25	10	
**Size**				
≥5 cm	59	44	15	1
<5 cm	23	18	5	
**Depth**				
deep	62	48	14	0.554
superficial	20	14	6	
**MVD**				
≥15/HPF	43	34	9	0.607
<15/HPF	39	28	11	
**MIB-1**				
positive (≥25.8%)	33	23	10	0.223
negative (<25.8%)	49	39	10	
**HIF-2α (*n* = 69)**				
positive	24	16	8	0.236
negative	45	35	10	
**Necrosis**				
No necrosis	38	32	6	
<50%	33	20	13	
≥50%	11	10	1	0.031*
**AJCC stage (*n* = 81)**				
I	24	18	6	0.301
II	40	30	10	
III	14	12	2	
IV	3	1	2	

Abbreviations: MVD = microvessel density; HPF = high power field.

*P <0.05.

### Immunohistochemistry

Immunohistochemical staining was performed as described previously [24, 25]. Sections were pretreated with Target Retrieval Solution (Dako Denmark A/S, Glostrup, Denmark) in a microwave oven at 100°C for 20 min before being incubated with anti-HIF-1α monoclonal antibodies (HIF-1 alpha; H1alpha67, 1:100 dilution; Novus Biologicals) at 4°C overnight. The immune complex was detected with the DAKO EnVision Detection System (Dako). The serial sections were also immunostained with anti-Ki-67 (MIB-1) antibody (M 7240, 1:100; Dako) and anti-CD31 antibody (JC70A, 1:20; Dako). HIF-1α staining was considered positive if ≥10% of tumor cell nuclei were stained [26]. The MIB-1 index and microvessel density (MVD) were calculated as described previously [27, 28]. The cut-off value of the MIB-1 index was calculated by receiver operating characteristic curve analysis of the MIB-1 index and overall survival. When the MIB-1 index was 25.8 or more, MIB-1 immunoreactivity was considered to be positive. The percentage of the tumor area exhibiting necrosis was evaluated as previously described [24]. Immunostaining of HIF-2α was also performed with anti-HIF-2α monoclonal antibodies (HIF-2α; ep190b, 1:1000; Abcam, Cambridge, UK) on 69 available specimens [29]. Immunohistochemical results were judged by two investigators (M. Endo and Y. Oda) who were blinded to the clinical status of the patients.

### Statistical analysis

All data analysis was conducted with the JMP10 statistical software package (SAS Institute, Cary, NC, USA). Data in graphs are presented mainly as box-and-whisker plots, and statistical comparisons were performed using the Mann-Whitney U test. The chi-square test or Fisher’s exact test was used as appropriate for two-group comparisons. Survival curves were calculated by the Kaplan-Meier method, and the differences were compared by the log-rank test. Hazard ratios for risk factors for death were evaluated using the Cox proportional hazards regression model. *P* <0.05 was considered as statistically significant.

### Cell lines

Human MPNST cell line FMS-1 was kindly provided by M. Hakozaki (First Department of Pathology, Fukushima Medical University School of Medicine, Fukushima, Japan) [30]; HS-Sch-2 was provided by the RIKEN BRC through the National Bio-Resource Project of the Ministry of Education, Culture, Sports, Science and Technology (MEXT), Japan; FU-SFT8611 and FU-SFT9817 were established by M. Aoki and H. Iwasaki (Department of Pathology, Fukuoka University School of Medicine, Fukuoka, Japan) [31]. FMS-1 cells were cultured in RPMI-1640; HS-Sch-2 was maintained in Dulbecco’s modified Eagle’s medium (DMEM); FU-SFT8611 and FU-SFT9817 were cultured in DMEM/F-12. Each medium was supplemented with 10% fetal bovine serum (FBS) (HyClone Laboratories, Inc., Logan, UT, USA), 100 units per ml penicillin, and 100 μg per ml streptomycin at 37°C in an atmosphere of 5% CO_2_. To produce a hypoxic culture condition, the BIONIX-3 hypoxic culture kit was used (Sugiyama-Gen, Tokyo, Japan). Short tandem repeat analysis was performed in MPNST cell lines (Takara Bio, Otsu, Japan). In HS-Sch-2, the evaluation value was 0.944, and the identity of the cell line in the database was confirmed. FMS-1, FU-SFT8611, and FU-SFT9817 were not registered in the database.

### Western blot analysis

Cytoplasmic and nuclear extractions of MPNST cell lines were prepared separately using NE-PER^®^ Nuclear and Cytoplasmic Extraction Reagents (Thermo Fisher Scientific, Waltham, MA, USA). To inhibit the degradation of HIF-1α overexpressed under hypoxic conditions for 24 h, the cells were scraped and collected immediately after removal from the hypoxic chamber. Cellular extractions stimulated with chetomin or deferoxamine (DFO) for 24 h were also collected. Western blot analysis was performed as described previously [32, 33] with the following primary antibodies: HIF-1α (1:1000, Abcam), β-actin (1:1000, Santa Cruz Biotechnology, Dallas, TX, USA), and lamin A/C (1:200, Santa Cruz). Immunoblotting of HIF-1α was performed with nuclear extraction as described previously [34, 35].

### Cell growth assay

To compare proliferation under normoxia and hypoxia, the aforementioned CellTiter-Glo^®^ Luminescent Cell Viability Assay was used again. For each cell line, 2 × 10^3^ cells were seeded into each well of a 96-well plate and cell viability was measured immediately and again after culturing for 48 h each under normoxic and hypoxic conditions; furthermore, the growth rates from 0 h were calculated. In the cell growth assay, each medium was supplemented with 1% FBS, 100 units per ml penicillin and 100 μg per ml streptomycin. The concentration of FBS in the cell growth assay was lowered to 1% in order to reduce the involvement of growth factors.

### siRNA experiments

Transfection of siRNA was carried out according to the manufacturer’s protocols. All four MPNST cell lines were seeded at 1 × 10^5^ cells per well in 6-well plates without antibiotics. After 24 h in culture, cells were transfected with HIF-1α siRNA (sc-35561, Santa Cruz Biotechnology) or control siRNA-A (sc-37007, Santa Cruz Biotechnology) using Lipofectamine^®^ 2000 (Thermo Fisher). The introduction of the siRNAs was confirmed by real-time quantitative polymerase chain reaction (PCR) and immunoblotting. Forty-eight hours after transfection, a growth assay, chemosensitivity assay, and cell cycle assay were performed as described above.

### Quantitative PCR

Real-time quantitative PCR was carried out using a LightCycler 1.5 (Perfect Real Time, Takara Bio), using the conditions described previously [36]. The primers specific to the genes of interest are summarized in Table 2.

**Table 2:** Primers used for real-time quantitative PCR.

Gene (Accession Number)		5'- Primer -3'
*HIF1A* (NM_001530.3)	Forward	TTCACCTGAGCCTAATAGTCC
	Reverse	CAAGTCTAAATCTGTGTCCTG
*GAPDH* (NM_002046.5)	Forward	TGTTGCCATCAATGACCCCTT
	Reverse	CTCCACGACGTACTCAGCG
*VEGFA* (NM_001171623.1)	Forward	GTCCCAGGCTGCACCCATG
	Reverse	AGGAAGCTCATCTCTCCTA
*GLUT1*(NM_006516.2)	Forward	GAGTTCTACAACCAGACATGG
	Reverse	GCATTGAATTCCGCCGGCCA
*BNIP3* (NM_004052.3)	Forward	CCGGGATGCAGGAGGAGAG
	Reverse	TTATAAATAGAAACCGAGGCTGGAAC
*CCND1* (NM_053056.2)	Forward	GCGCGCCCTCGGTGTCCTA
	Reverse	GCGACAGGAAGCGGTCCAGG

The expression of mRNA was calculated using LightCycler version 3.5 software (Roche Diagnostics), and data were standardized against those of the GAPDH housekeeping gene. Assays were performed in triplicate.

### Apoptosis and cell cycle analysis by flow cytometry

Each MPNST cell line (1 × 10^5^/well) was seeded in a 60-mm dish with various concentrations of drugs. Flow cytometric analyses were performed with the BD Accuri C6 flow cytometer (Becton, Dickinson and Company, Franklin Lakes, NJ, USA). After culturing for 12 h, cell apoptosis was analyzed with the Annexin V-FITC/7-AAD Kit (Beckman Coulter, Brea, CA, USA). Cell cycle analysis used cells cultured for 48 h with various concentrations of chetomin. Experimental procedures were described previously [33].

### Inhibitor assay

We used an inhibitor kit provided by the Screening Committee of Anticancer Drugs (SCADS Inhibitor Kit) with the support of a Grant-in-Aid for Scientific Research on Innovative Areas, Scientific Support Programs for Cancer Research. The screening kit consisted of different 362 compounds. A total of 2 × 10^3^ cells were incubated without any compound for the first 16 h and then with each inhibitor at the concentration of 1μM for the next 48 h. Each compound was judged effective if the cell survival rate, as assessed by the CellTiter-Glo^®^ Luminescent Cell Viability Assay (Promega, Madison, WI, USA), was less than 20% as compared with dimethyl sulfoxide (DMSO). The CellTiter-Glo^®^ experimental procedure was described previously [32]. This screening assay was performed in duplicate.

### Reagents

Chetomin was obtained from BioViotica Naturstoffe GmbH (Göttingen, Germany) and diluted in DMSO. Deferoxamine mesylate (DFO) was purchased from BioVision-Life Science Source (Milpitas, CA, USA) and dissolved in distilled water. Although DFO is classically used to create a pseudo-hypoxic environment, in this study it was used only to obtain the positive control of HIF-1α in Western blot analyses [37].

### Chemosensitivity assay

The chemosensitivity assay was performed using the CellTiter-Glo^®^ Luminescent Cell Viability Assay. For assays in a hypoxic environment, 2 × 10^3^ cells were incubated with various concentrations of drugs under normoxia for the first 16 h and then under hypoxia for the next 48 h. Each experiment was conducted in triplicate or more.

### Generation of stable HIF reporter MPNST cell lines and reporter assay

HeLa cells that were stably transfected with an HIF luciferase reporter were obtained from Signosis (Santa Clara, CA, USA). Other stably transfected MPNST cell lines, namely FMS-1/HIF-luc, HS-Sch-2/HIF-luc, and FU-SFT8611/HIF-luc, were generated with the Cignal Lenti HIF reporter (luc) (Qiagen, Hilden, Germany). Lentiviral transfections and puromycin selections were performed according to the manufacturer’s protocol. Luciferase activity was measured 12 h after the addition of each reagent using the Dual-Luciferase reporter assay system (Promega, Madison, WI, USA). Each experiment was conducted in triplicate.

## Results

### Association of nuclear HIF-1α expression and clinical MPNST outcomes

**Fig 1:**
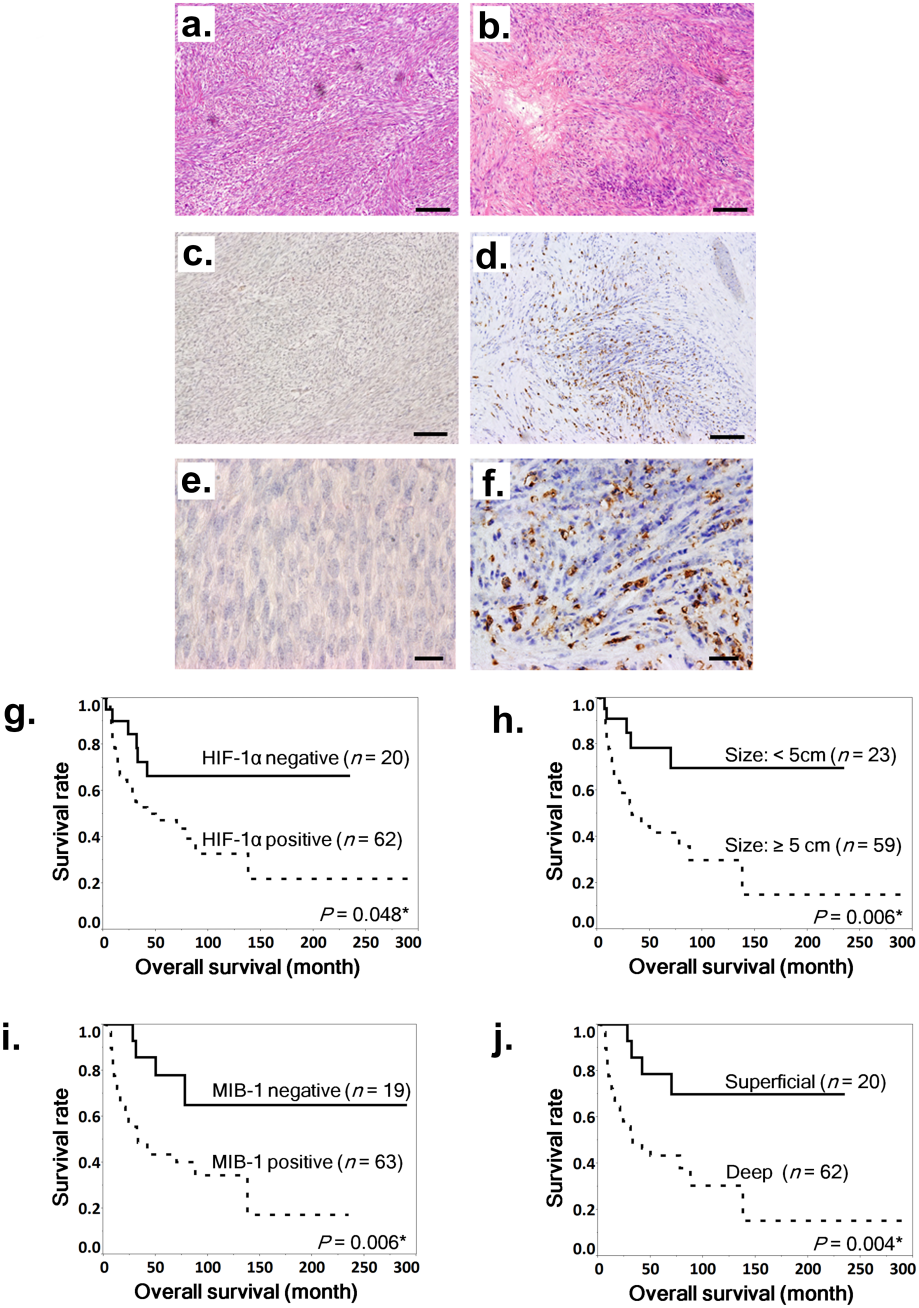
Hematoxylin and eosin staining and immunohistochemical staining of HIF-1α in MPNST samples and association between nuclear HIF-1α expression and poor prognosis in MPNSTs. **a**–**f**. Staining with hematoxylin and eosin (a and b) and immunohistochemical staining of HIF-1α (c–f) in MPNST specimens. Representative cases: a HIF-1α–negative specimen (a, c, and e) and a HIF-1α–positive specimen (b, d, and f). Scale bar, 100 μm in a–d and 20 μm in e and f. **g**–**j**. Kaplan-Meier survival curves for all patients based on positive or negative nuclear HIF-1α expression (g), tumor diameter of 5 cm or more (h), positive or negative MIB-1 expression (i), and deep tumor location (j). Log-rank tests were performed to determine statistical significance.

We first examined the clinical significance of HIF-1α in MPNST. The HIF-1α positivity rate of clinical specimens was 75.6% (62/82 cases) (Fig 1a–1f). In the log-rank test, deep tumor location, MIB-1 positivity, large tumor size (≥5 cm), and HIF-1α positivity were significant poor prognostic factors (Fig 1g–1j).

On the other hand, the HIF-2α positivity rate was 34.8% (24/69 cases) (Fig a–d in S1 Fig), and HIF-2α positivity was not a significant prognostic factor (Fig e in S1 Fig). Tumor necrosis also did not significantly correlate with poor prognosis (Fig f in S1 Fig). In univariate Cox analysis, large tumor size (*P* = 0.034), deep tumor location (*P* = 0.002), MIB-1 positivity (*P* = 0.003), AJCC stage, and HIF-1α positivity (*P* = 0.038) were significantly associated with poor prognosis; however, prognosis was not significantly associated with NF1 status, MVD, age, sex, tumor location, HIF-2α positivity, or tumor necrosis (Table 3). Furthermore, multivariate analysis demonstrated that HIF-1α positivity was an independent prognostic factor (*P* < 0.0001), as was MIB-1 positivity (*P* < 0.0001), percentage necrotic area >50% (*P* = 0.039), and AJCC stage (*P* = 0.0036) (Table 3). HIF-1α positive specimens showed significantly more cases with >50% necrosis, but there was no significant association between HIF-1α and the status of the other parameters (Table 1). These results suggested that HIF-1α plays an important role in MPNST, and we therefore conducted subsequent experiments with MPNST cell lines.

**Table 3:** Results of univariate and multivariate analyses for overall survival.

variable	Univariate analysis		Multivariate analysis	
	HR (95%CI)	*P* value	HR (95%CI)	*P* value
**NF1**				
present absent	1.48 (0.74–2.82)	0.273		
	1			
**HIF-1α**				
positive negative	2.35 (1.04–6.28)	0.038*	8.26 (2.76–28.85)	<0.0001*
	1		1	
**Age**				
≥50 <50	0.76 (0.38–1.47)	0.417		
	1			
**Sex**				
male female	1.44 (0.74–2.84)	0.282		
	1			
**Location**				
trunk extremities	1.90 (0.96–4.04)	0.065	1.43 (0.66–3.23)	0.367
	1		1	
**Size**				
≥5 cm <5 cm	3.46 (1.46–10.19)	0.034*	2.27 (0.66–9.95)	0.207
	1		1	
**Depth**				
deep superficial	4.07 (1.59–13.76)	0.002*	0.91 (0.23–4.08)	0.896
	1		1	
**MVD**				
≥15/HPF <15/HPF	1.88 (0.96–3.82)	0.066		
	1			
**MIB-1**				
positive negative	5.59 (2.70–12.23)	<0.0001*	10.16 (3.60–32.05)	<0.0001*
	1		1	
**HIF-2α (*n* = 69)**				
positive negative	1.16 (0.50–2.48)			
	1			
**Necrosis**				
No <50% ≥50%	1		1	
	1.82 (0.90–3.82)	0.098	1.93	0.187
	2.05 (0.65–5.50)	0.201	4.39	0.039*
**AJCC stage (*n* = 81)**				
I II III IV	1		1	
	2.61 (1.11–7.16)	0.028*	1.25 (0.41–4.20)	0.706
	2.53 (0.79–8.12)	0.116	0.27 (0.06–1.29)	0.101
	35.2 (6.63–160.8)	0.0002*	17.51 (2.79–100.40)	0.0036*

Abbreviations: HR = hazard ratio; CI = confidence interval.

*P <0.05.

### Nuclear expression of HIF-1α in MPNST cell lines under normoxia and hypoxia

**Fig 2:**
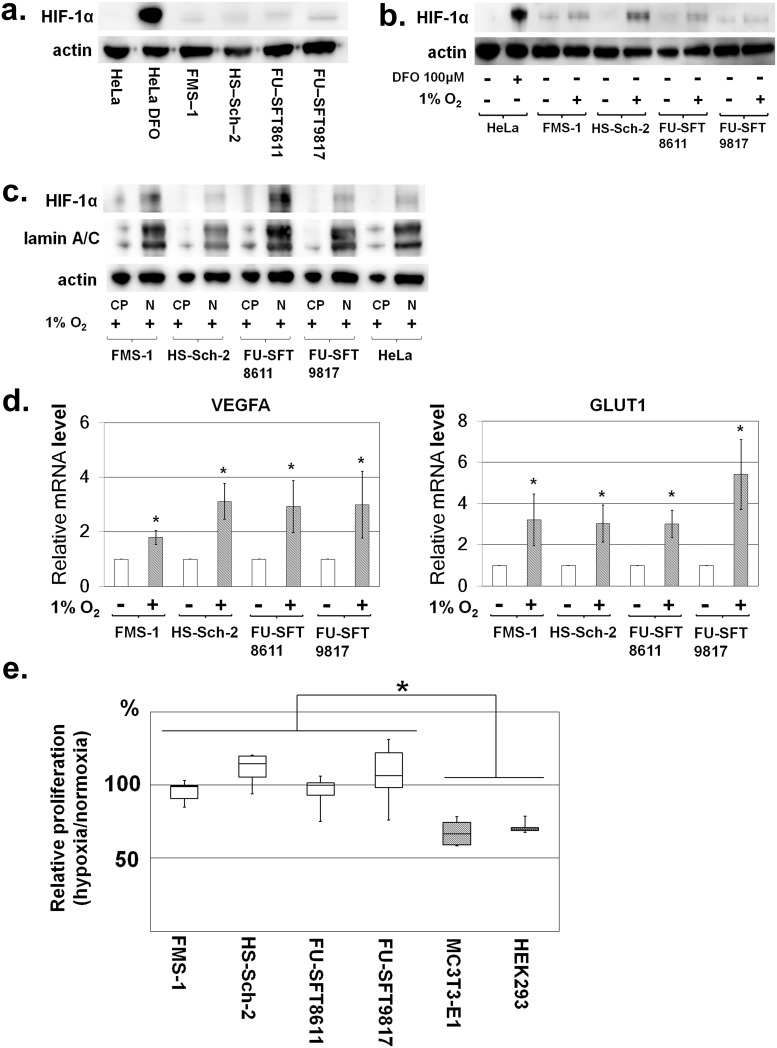
Nuclear expression of HIF-1α and MPNST cell line proliferation under normoxic and hypoxic conditions. **a**. Nuclear expression of HIF-1α in MPNST cell lines under normoxia was confirmed by Western blotting of HIF-1α using nuclear extract from each cell line. In HeLa (cervical cancer cell line) cells, the expression of HIF-1α is shown under hypoxia caused by the addition of DFO. Meanwhile, definite expression of HIF-1α was observed in all MPNST cell lines. **b**. Nuclear expression of HIF-1α in MPNST cell lines was induced by hypoxia. Actin was used for internal normalization. **c**. Based on comparison with nuclear protein lamin A/C, HIF-1α was mainly localized in the nuclei. In Fig 2c, “CP” represents cytoplasmic proteins, and “N” represents nuclear proteins. **d**. VEGFA and GLUT1 expression downstream of HIF-1α was enhanced under hypoxia. **e**. Cell proliferation under normoxic and hypoxic conditions in MPNST cell lines. Growth at 48 h after cell seeding was compared under normoxia and hypoxia. In the hypoxic condition, cell proliferation of non-transformed cell lines (MC3T3-E1 and HEK293) was suppressed; however, growth of MPNST cell lines under hypoxia was comparable to that under normoxia. Experiments were performed six times. Data in graphs are presented as box-and-whisker plots, and statistical comparisons were performed using the Mann-Whitney U test. *P <0.05.

We investigated the expression levels of HIF-1α in four human MPNST cell lines to confirm our histological results in human MPNST samples. We found that in MPNST cell lines, the constitutive weak expression of nuclear HIF-1α was observed even under normoxia (Fig 2a). Since oxygen concentrations in tumors are considered to be lower than those in normal tissues [38], we further evaluated HIF-1α expression under hypoxia in MPNST cell lines. Cultures with 1% oxygen concentration were prepared using the hypoxic chamber, and this hypoxic condition increased the expression of HIF-1α (Fig 2b). Furthermore, HIF-1α localization was determined (Fig 2c) by comparing its nuclear distribution with that of lamin A/C. VEGFA and GLUT1 expression, which are downstream genes of HIF-1α, was also enhanced in the hypoxic environment, reflecting the up-regulation of HIF-1α (Fig 2d).

### Specific suppression of HIF-1α by siRNA reduced proliferation of MPNST cell lines and induced apoptosis in a hypoxic environment

Hypoxia-induced overexpression of HIF-1α in MPNST cells might affect the proliferative phenotype of tumor cells; thus, we next compared the proliferation of normal and MPNST cell lines under hypoxic and low-serum culture conditions. Hypoxia reduced the proliferation of MC3T3-E1 and 293 cells (reductions of 32.7% ± 8.95% and 29.0% ± 3.97%, respectively) but not MPNST cell lines (Fig 2e). MPNST cell lines showed high adaptability to low serum and hypoxia, suggesting that cellular adaptation to hypoxic stress in MPNST cells is induced by HIF-1α. To test this hypothesis, siRNA was used to specifically inhibit HIF-1α in MPNST cells.

**Fig 3:**
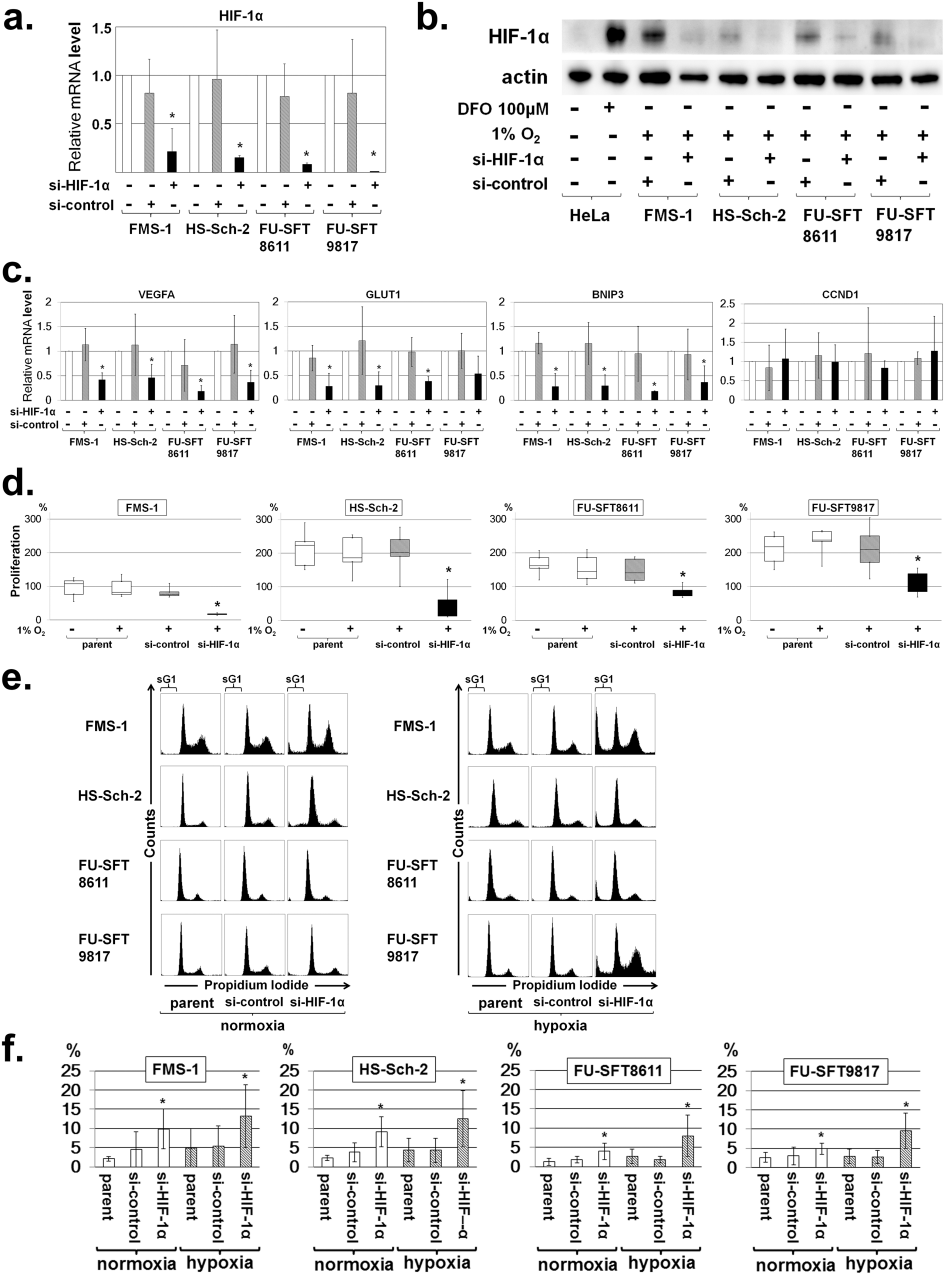
Knockdown of HIF-1α by si-RNA and effects of si-HIF-1α on cell proliferation and cell cycle progression. Assessment of HIF-1α knockdown in MPNST cell lines by the following: **a**. Real-time quantitative PCR (mean ± SD; *P < 0.05) under normoxia; and **b**. Western blotting of HIF-1α after induction of HIF-1α–specific si-RNA under hypoxia. The si-HIF-1α suppressed the expression of HIF-1α compared to control si-RNA. Actin was used for internal normalization. **c**. Downregulation of downstream genes by si-HIF-1α. VEGFA, GLUT1, and BNIP3 expression downstream of HIF-1α was decreased by si-HIF-1α, whereas expression of CCND1 downstream of HIF-2α remained unchanged. **d**. Effects of si-HIF-1α on cell proliferation in MPNST cell lines. Knockdown of HIF-1α by si-RNA suppressed the proliferation of MPNST cell lines under hypoxia. Data in graphs are presented as box-and-whisker plots, and statistical comparisons were performed using the Mann-Whitney U test. *P < 0.05. **e and f**. Effects of si-HIF-1α on cell cycle progression in MPNST cell lines. **e**. Representative cell cycle profile of MPNST cell lines after knockdown of HIF-1α by si-RNA. The areas labelled “sG1” in the figures represent subG1 fractions. **f**. SubG1 fractions increased by HIF-1α knockdown in MPNST cell lines under normoxia and hypoxia. Experiments were performed in triplicate or more, and data are expressed as the mean ± SD. *P < 0.05. Each value is listed in S1 Table.

Marked knockdown of HIF-1α mRNA was observed 48 h after transfection under hypoxia (Fig 3a). This construct also reduced the expression of nuclear HIF-1α protein under hypoxia (Fig 3b). As the expression of HIF-1α decreased, VEGFA, GLUT1, and BNIP3, all of which are downstream genes of HIF-1α, demonstrated down-regulated mRNA expression in real-time PCR (Fig 3c). The si-RNA–induced knockdown of HIF-1α suppressed the proliferation of MPNST cell lines in hypoxia (Fig 3d). Remarkably, flow cytometer analysis with propidium iodide (PI) staining at 48 h after transfection revealed a significant increase of subG1 fractions in MPNST cells under hypoxia (Fig 3e and 3f, S1 Table). Taken together, these studies showed the critical role of HIF-1α in MPNST cell growth and survival, thereby supporting the rationale for further investigation of anti-HIF-1α therapeutic strategies in MPNST.

### Chetomin, an inhibitor of HIF-1α/p300 interaction, exhibited anti-tumor activity and induced apoptosis in MPNST cell lines

**Fig 4:**
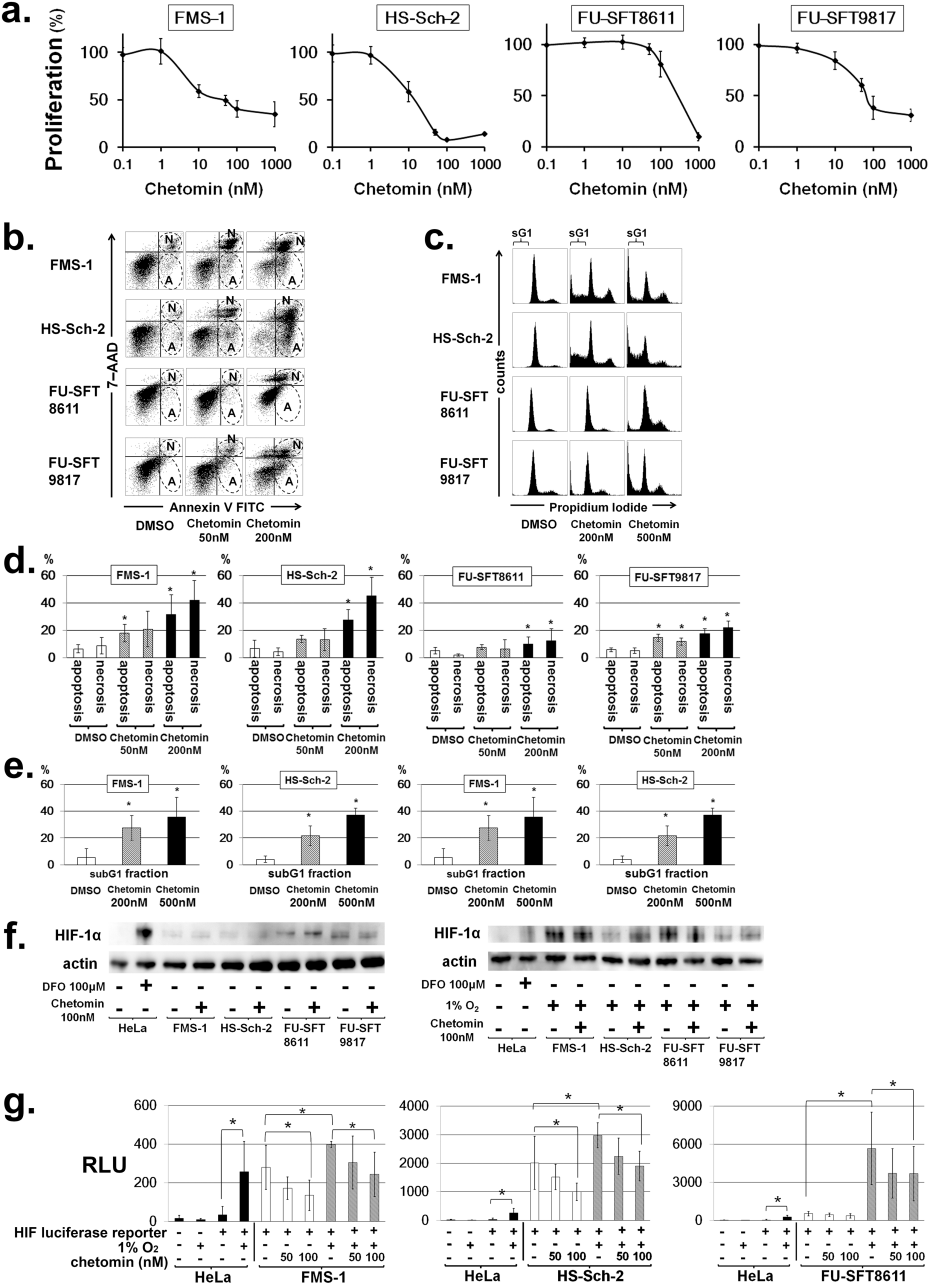
Chetomin, an inhibitor of HIF-1α/p300 interaction, effectively inhibited the growth of MPNST cells and induced their apoptosis by attenuating the transcriptional activity of HIF-1. **a**. Effects of chetomin on cell proliferation in MPNST cell lines. Chetomin inhibited the cell proliferation of MPNST cell lines under hypoxic conditions. Data are expressed as the mean ± SD. *P < 0.05. **b**. Effects of chetomin on apoptosis in MPNST cell lines. Twelve hours after the addition of chetomin, double staining with annexin V FITC and 7-AAD was performed, and apoptosis was analyzed by flow cytometer. An increase in apoptotic and necrotic fractions was observed in all cell lines in a dose-dependent manner. The early apoptotic component is annexin V–positive and 7-AAD–negative, corresponding to the lower right quadrant of each panel. On the other hand, the necrotic component is positive for both annexin V and 7-AAD, and is indicated by the upper right quadrant of each panel. The areas surrounded by broken lines and labelled “A” represent apoptotic fractions, while those labelled “N” correspond to necrotic fractions. **c**. Representative cell cycle profile of MPNST cell lines after treatment of chetomin. The areas labelled “sG1” represent subG1 fractions. **d and e**. Chetomin increases apoptosis and subG1 fractions in MPNST cell lines. Experiments were performed in triplicate, and data are expressed as the mean ± SD. *P < 0.05. Each value is provided in S3 and S4 Tables. **f**. Effects of chetomin on the nuclear expression of HIF-1α. MPNST cell lines were treated with chetomin and the nuclear expression of HIF-1α was evaluated by Western blotting. Actin was used for internal normalization. The nuclear expression of HIF-1α was not affected by chetomin, even under normoxia or hypoxia. **g**. Hypoxia-inducible reporter assay. MPNST cell lines were transfected with a luciferase HIF reporter. Reporter activities under normoxic or hypoxic conditions and in the absence or presence of the indicated concentrations of chetomin were normalized to an internal control and expressed as relative light units (RLUs). Experiments were performed in triplicate, and data are expressed as the mean ± SD. *P < 0.05.

We conducted comprehensive screening with about 400 chemicals from the SCADS Inhibitor Kit (provided by the Screening Committee of Anticancer Drugs, Japan). These compounds, all of which have known targets, consist of signaling pathway inhibitors, kinase inhibitors, metabolic pathway inhibitors, and classical anti-cancer agents. This method showed that eight compounds were effective against more than one cell line (S2 Table). Significantly, chetomin, a compound that inhibits the transcriptional activity of HIF-1α by impeding the binding between HIF-1α and the p300/CBP complex [39], exhibited more than 80% growth inhibition at 1 μM in three of four MPNST cell lines. Thus, we proceeded with subsequent experiments using chetomin. First, we confirmed the growth inhibitory effect of chetomin at low concentration in MPNST cell lines in a normoxic environment. Since we already showed that the expression of HIF-1α was elevated under hypoxia in MPNST cells (Fig 2b), we next investigated the effect of chetomin in a hypoxic environment and found that MPNST cell line proliferation was suppressed (Fig 4a). Chetomin increased cell apoptosis in a dose-dependent manner (Fig 4b and 4d, S3 Table) and also increased the subG1 fraction (Fig 4c and 4e, S4 Table). Overall, chetomin showed cytocidal effects on MPNST cell lines.

### Chetomin attenuates the transcriptional activity of HIF-1α

Chetomin reduces the transcriptional activity of HIF by inhibiting the interaction between HIF-1α and p300 in the nucleus, and does not affect the amount of HIF-1α [39]. We found that the addition of chetomin did not reduce the nuclear expression of HIF-1α (Fig 4f), which supports the known mechanism of chetomin inhibiting the binding of HIF-1α and p300 rather than suppressing the expression of HIF-1α itself. We then used MPNST cells stably transfected with the HIF reporter to determine whether HIF-1α reporter activity was inhibited by chetomin. A hypoxic environment enhanced reporter activity, and chetomin at nanomolar concentrations effectively inhibited reporter activity in a dose-dependent manner (Fig 4g). Interestingly, reporter activity in MPNST cell lines tended to be higher than in HeLa cells under normoxic conditions, as shown in Fig 2a, a finding that was compatible with the constitutive and hypoxia-independent expression of HIF-1α in MPNST cell lines. Chetomin also reduced reporter activity under normoxia in FMS-1/HIF-luc and HS-Sch-2/HIF-luc but not FU-SFT8611/HIF-luc. Furthermore, VEGFA and GLUT1 expression, which are downstream genes of HIF-1α, was decreased by chetomin. Although the expression of VEGFA in FU-SFT8611 cells was decreased, there was no statistically significant difference (S2 Fig). Overall, these results showed that chetomin attenuated HIF-1α–mediated transcription and that this effect might be associated with the anti-tumor activity of chetomin in MPNST cells; that is, chetomin was a specific rather than global inhibitor of the HIF-1α signaling pathway at the concentrations used in these experiments.

## Discussion

HIF-1α has been proven to contribute to poor prognosis and to play a critical role in tumor metastasis and angiogenesis in many types of solid tumors, including renal cell carcinoma [40] and lung cancer [18]. In addition, a recent meta-analysis of the prognostic significance of HIF-1α in patients with bone and soft tissue sarcoma showed that nuclear expression of HIF-1α was significantly associated with poorer disease-free and overall survival [21]. However, that study used a heterogeneous group of patients and thus the exact role of HIF-1α in MPNSTs remains unknown. In this study, we conducted a clinicopathological and prognostic analysis of nuclear HIF-1α expression in 82 MPNST clinical specimens. We consider that our univariate and multivariate analyses provide the first evidence that the nuclear expression of HIF-1α is associated with poor overall survival in patients with MPNSTs. Importantly, the nuclear expression of HIF-1α was not correlated with MVD, suggesting that HIF-1α might exert its function in a cell-autonomous manner rather than by stimulating tumor angiogenesis in MPNSTs.

Characterizing the constitutive expression of HIF-1α under normoxia was one of the hallmarks of this study. Under normoxia, HIF-1α undergoes quick degradation and the half-life of HIF-1α is very short (about 5 min) [41]. Therefore, even in tumor cells such as those of the HeLa cell line, the expression of HIF-1α was not detected under normoxia (Fig 2a and 2b). However, we observed positive expression of HIF-1α in MPNST cells in a normoxic environment. Previous reports [42] showed that activating the Akt/mTOR pathway increased the rate of mRNA translation into HIF-1α protein, and we already reported that the Akt/mTOR pathway was activated in MPNSTs [24]. Thus, we demonstrated that the HIF-1α protein is produced more rapidly than it is degraded in MPNST cells. For accurate evaluation of intracellular HIFs in future research, reconsideration will be necessary for the extracting method of protein. The use of the ultrasonic homogenizer is one of the promising methods [43].

Identification of the cell-autonomous roles of HIF-1α in MPNSTs is the next issue to be addressed. HIF-1α usually takes part in adaptive responses under hypoxia to promote or maintain tumor cell survival, and this was true in MPNST cells as shown in Fig 3c and 3d. Recently, a role for HIF-1α in regulating apoptosis was also proposed [44, 45]. For example, specific down-regulation of HIF-1α by RNA interference significantly enhanced apoptosis under hypoxia by preventing the hypoxia-mediated increase in GLUT-1 in Ewing's sarcoma and rhabdomyosarcoma [44]. Our immunohistochemical results also support the idea that the tumor-promoting function of HIF-1α is an anti-apoptotic effect of MPNST cells rather than a proliferative one, though these results could not demonstrate that the correlation between HIF-1α positivity and MIB-1 expression reflected the proliferative ability of the tumor cells. In this study, we showed that the knockdown of HIF-1α in MPNSTs enhanced apoptosis under hypoxia. We investigated the underlying mechanism of this process. Well-known downstream genes such as VEGFA and GLUT1 were down-regulated by inhibition of HIF-1α, but further detailed experiments should also be performed about the expressions of other genes related to HIF-1α in the future.

Our clinical and experimental data strongly suggested that HIF-1α was associated with anti-apoptosis and poor survival in MPNSTs. A previous study of MPNST suggested the importance of HIF-1α in vitro [46], which supported our results. Thus, HIF-1α signaling is likely to be an attractive therapeutic target in MPNSTs. In this study, we attempted to identify new target molecules for inhibiting HIF-1α signaling in MPNSTs through cell-based screening using the SCADS Inhibitor Kit [47]. The advantage of this approach is that the SCADS Kit contains drugs whose pharmacokinetic, pharmacodynamic, and toxicity profiles are well known. We found that chetomin, an inhibitor of HIF-1α/p300 interaction, effectively inhibited MPNST cell growth and induced their apoptosis by attenuating the transcriptional activity of HIF-1α.

In this study, we performed a comprehensive analysis of primary MPNST cases that clearly demonstrated the negative correlation between the nuclear expression of HIF-1α and prognosis. We also clarified that inhibition of HIF-1α signaling suppressed growth and caused apoptosis in MPNST cells. Thus, we believe that the status of HIF-1α nuclear expression will provide useful prognostic information in patients with MPNSTs, and that HIF-1α signaling is a promising molecular target for novel therapeutic agents for MPNSTs.

## Supporting information

S1 Fig HIF-2α positivity and tumor necrosis did not correlate with prognosis. **a**–**d**. Immunohistochemical staining of HIF-2α in MPNST specimens. Representative cases: a HIF-2α–negative specimen (a, c) and a HIF-2α–positive specimen (b, d). Scale bar, 100 μm in a and b and 20 μm in c and d. **g**–**j**. Kaplan-Meier survival curves for all patients based on positive or negative nuclear HIF-2α expression (e) and degree of tumor necrosis (f). Log-rank tests were performed to determine statistical significance.
(TIF)

S2 Fig Downregulation of downstream genes of HIF-1α by chetomin. (a) Chetomin decreased the expression of VEGFA, GLUT1, and BNIP3 downstream of HIF-1α. The expression of VEGFA in FU-SFT 8611 was not significantly suppressed.
(TIF)

S1 Table SubG1 fractions markedly increased by HIF-1α knockdown in MPNST cell lines under hypoxia. The flow cytometer analysis with propidium iodide staining at 48 h after transfection of si-HIF-1α revealed a significant increase of subG1 fractions in MPNST cells under hypoxia. Experiments were performed in triplicate or more, and data are expressed as the mean ± SD. *P < 0.05.
(DOCX)

S2 Table In the screening kit consisting of different 362 compounds, eight compounds were effective against more than one MPNST cell line. In the inhibitor kit provided by the Screening Committee of Anticancer Drugs (SCADS), eight compounds added at a concentration of 1 μM markedly inhibited the proliferation of more than one MPNST cell line compared with DMSO. In particular, chetomin, an inhibitor of HIF-1α, showed efficacy against three MPNST cell lines, and this result was consistent with the proliferation inhibitory effect exhibited by si-HIF-1α.
(DOCX)

S3 Table Chetomin induced cell death of MPNST cell lines in a dose-dependent manner. As the results of apoptosis analysis by flow cytometry using Annexin V-FITC and 7-AAD, chetomin significantly increased both apoptotic and necrotic fractions compared to DMSO in four MPNST cell lines.
(DOCX)

S4 Table Induction of cell death of MPNST cell line by chetomin was also confirmed by cell cycle analysis. As the concentration of chetomin increased, cell cycle analysis with flow cytometer by propidium iodide staining revealed significant increase of subG1 fractions. These results suggested that chetomin induced cell death in the MPNST cell lines in a dose-dependent manner.
(DOCX)
