# Correction: Hypoxia-inducible factor 1 alpha is a poor prognostic factor and potential therapeutic target in malignant peripheral nerve sheath tumor

**DOI:** 10.1371/journal.pone.0194508

**Published:** 2018-03-15

**Authors:** Suguru Fukushima, Makoto Endo, Yoshihiro Matsumoto, Jun-ichi Fukushi, Tomoya Matsunobu, Ken-ichi Kawaguchi, Nokitaka Setsu, Keiichiro IIda, Nobuhiko Yokoyama, Makoto Nakagawa, Kenichiro Yahiro, Yoshinao Oda, Yukihide Iwamoto, Yasuharu Nakashima

Fig 4 is incorrect. The authors have provided a corrected version here.

**Fig 4:**
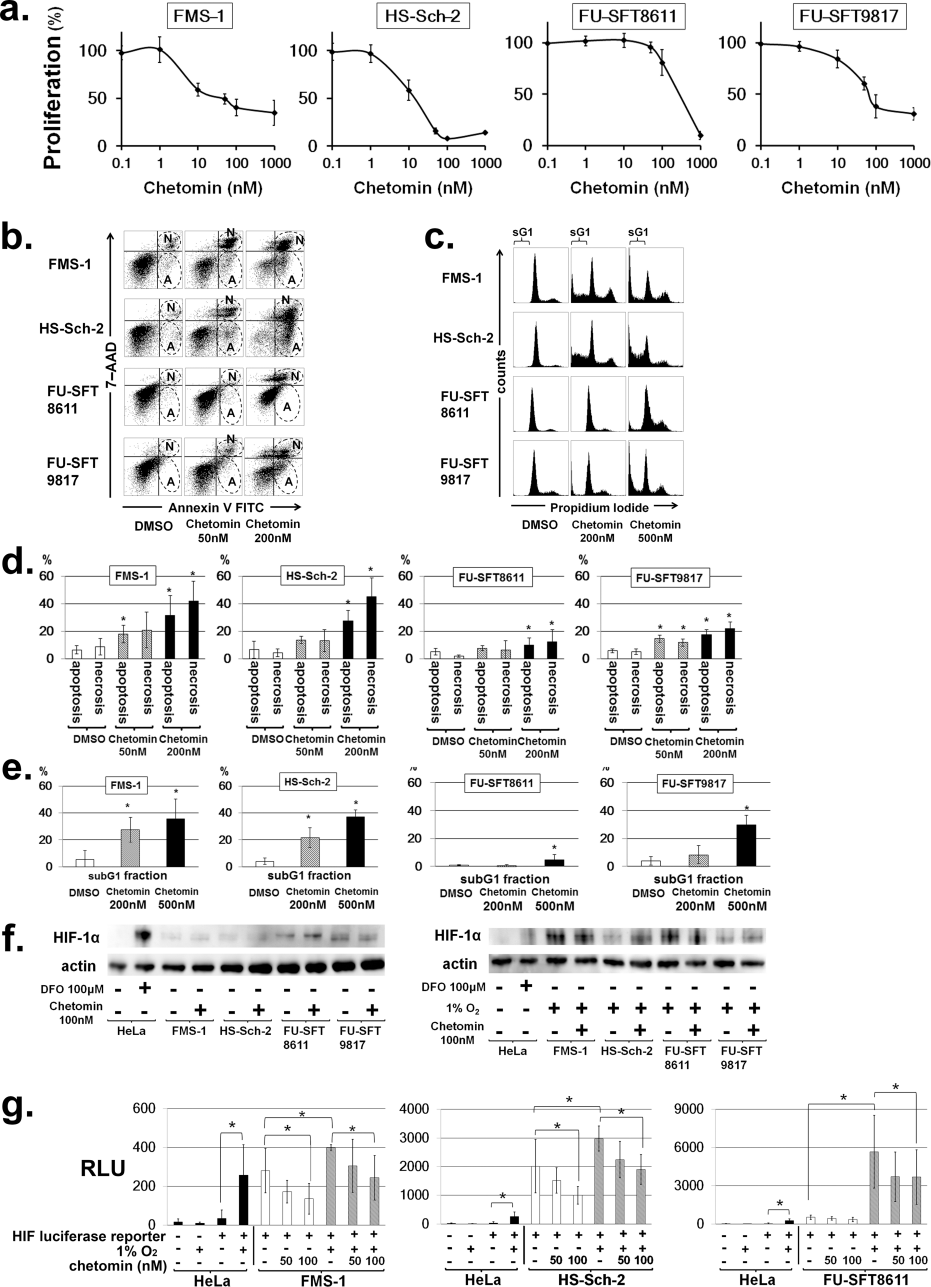
Chetomin, an inhibitor of HIF-1α/p300 interaction, effectively inhibited the growth of MPNST cells and induced their apoptosis by attenuating the transcriptional activity of HIF-1. a. Effects of chetomin on cell proliferation in MPNST cell lines. Chetomin inhibited the cell proliferation of MPNST cell lines under hypoxic conditions. Data are expressed as the mean ± SD. ^*^P < 0.05. b. Effects of chetomin on apoptosis in MPNST cell lines. Twelve hours after the addition of chetomin, double staining with annexin V FITC and 7-AAD was performed, and apoptosis was analyzed by flow cytometer. An increase in apoptotic and necrotic fractions was observed in all cell lines in a dose-dependent manner. The early apoptotic component is annexin V–positive and 7-AAD–negative, corresponding to the lower right quadrant of each panel. On the other hand, the necrotic component is positive for both annexin V and 7-AAD, and is indicated by the upper right quadrant of each panel. The areas surrounded by broken lines and labelled “A” represent apoptotic fractions, while those labelled “N” correspond to necrotic fractions. c. Representative cell cycle profile of MPNST cell lines after treatment of chetomin. The areas labelled “sG1” represent subG1 fractions. d and e. Chetomin increases apoptosis and subG1 fractions in MPNST cell lines. Experiments were performed in triplicate, and data are expressed as the mean ± SD. ^*^P < 0.05. Each value is provided in S3 and S4 Tables. f. Effects of chetomin on the nuclear expression of HIF-1α. MPNST cell lines were treated with chetomin and the nuclear expression of HIF-1α was evaluated by Western blotting. Actin was used for internal normalization. The nuclear expression of HIF-1α was not affected by chetomin, even under normoxia or hypoxia. g. Hypoxia-inducible reporter assay. MPNST cell lines were transfected with a luciferase HIF reporter. Reporter activities under normoxic or hypoxic conditions and in the absence or presence of the indicated concentrations of chetomin were normalized to an internal control and expressed as relative light units (RLUs). Experiments were performed in triplicate, and data are expressed as the mean ± SD. ^*^P < 0.05.
