# Supplementary figures and images for: Hypoxia-inducible factor 1 alpha is a poor prognostic factor and potential therapeutic target in malignant peripheral nerve sheath tumor
Source: PLoS One. 2017 May 30;12(5):e0178064. doi: 10.1371/journal.pone.0178064 (PMC5448771; doi:10.1371/journal.pone.0178064)

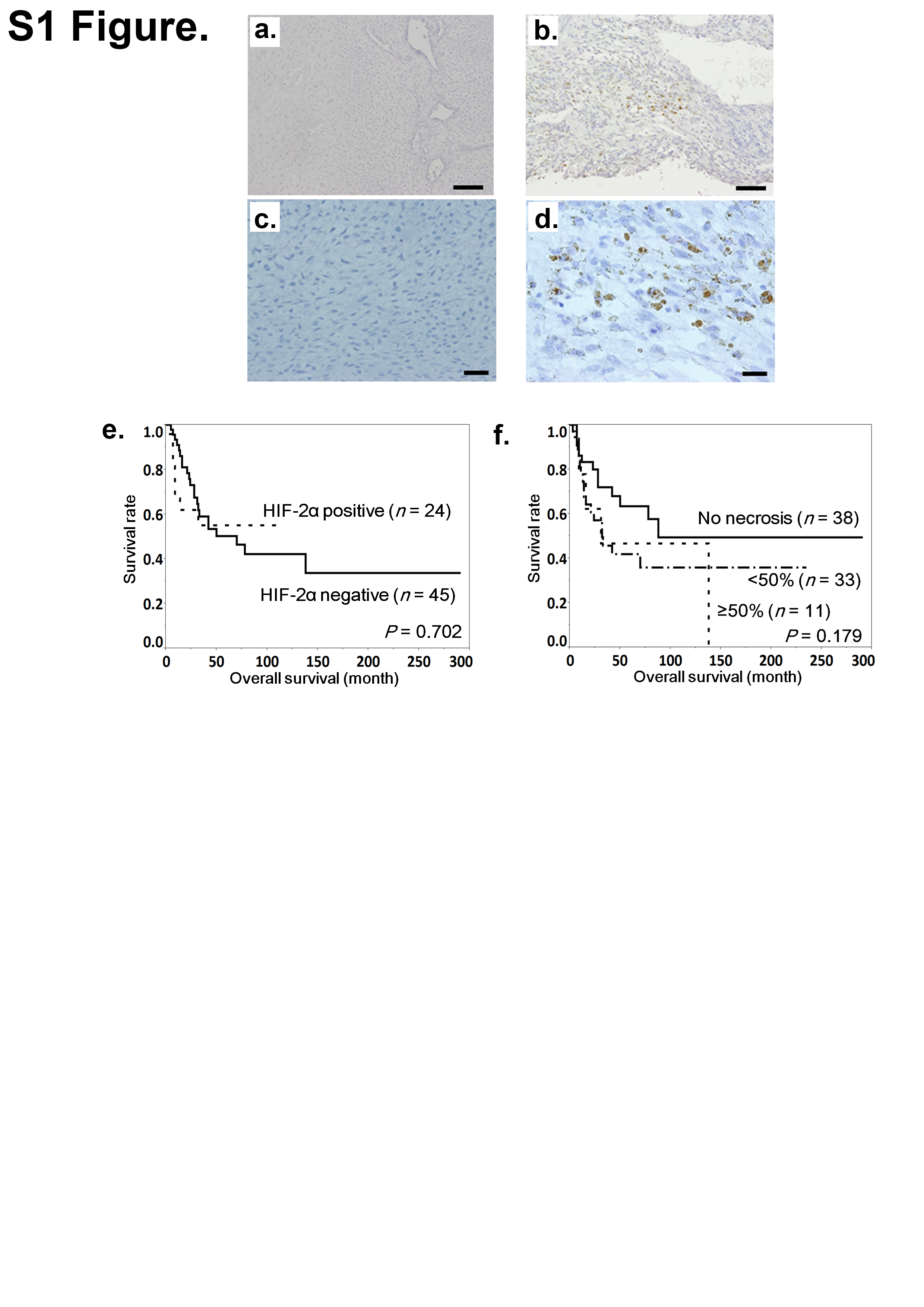

Supplement: S1 Fig — a–d. Immunohistochemical staining of HIF-2α in MPNST specimens. Representative cases: a HIF-2α–negative specimen (a, c) and a HIF-2α–positive specimen (b, d). Scale bar, 100 μm in a and b and 20 μm in c and d. g–j. Kaplan-Meier survival curves for all patients based on positive or negative nuclear HIF-2α expression (e) and degree of tumor necrosis (f). Log-rank tests were performed to determine statistical significance. (TIF) [file pone.0178064.s001.tif]

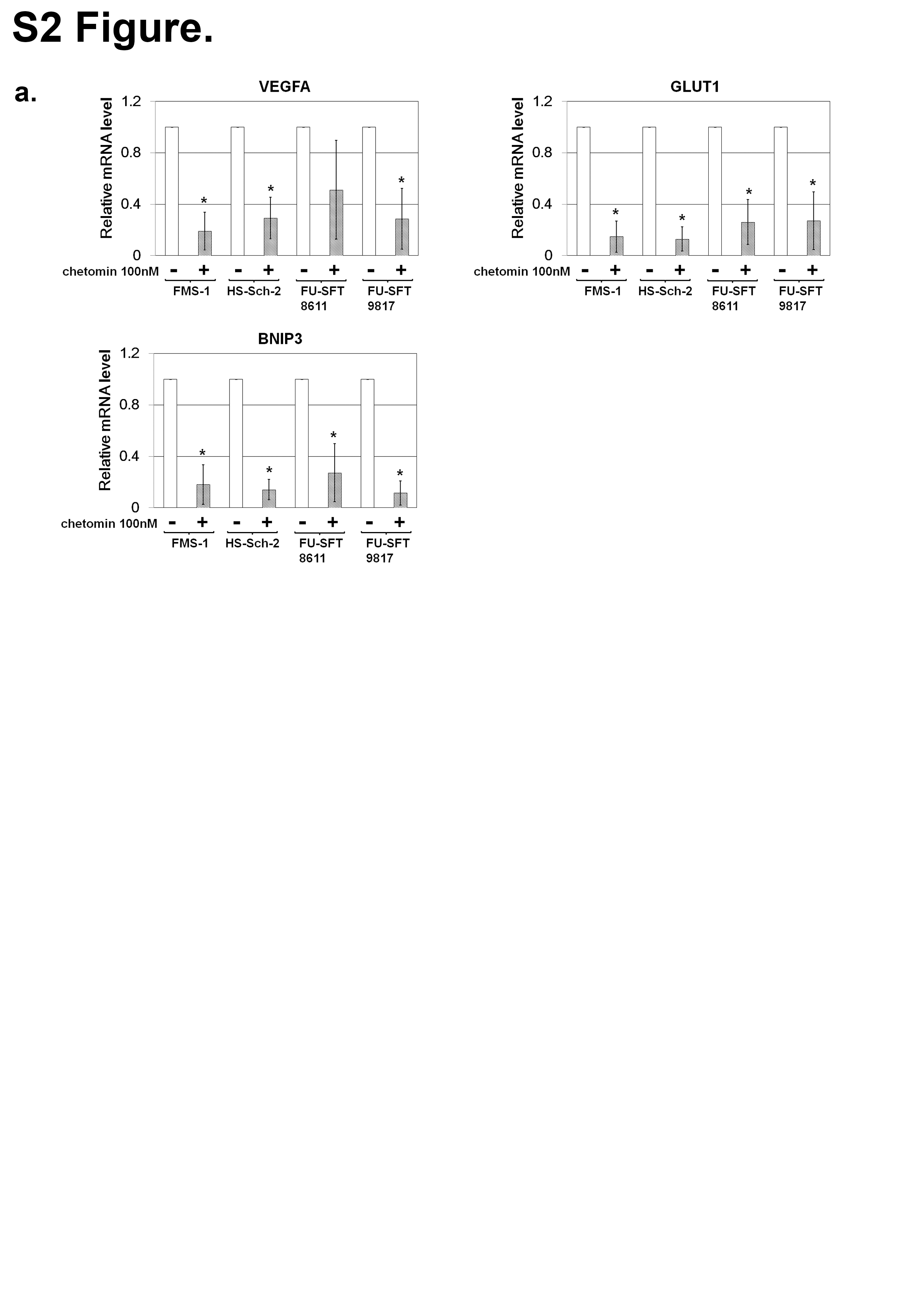

Supplement: S2 Fig — (a) Chetomin decreased the expression of VEGFA, GLUT1, and BNIP3 downstream of HIF-1α. The expression of VEGFA in FU-SFT 8611 was not significantly suppressed. (TIF) [file pone.0178064.s002.tif]
