# Supplementary material for: Hypoxia-inducible factor 1 alpha is a poor prognostic factor and potential therapeutic target in malignant peripheral nerve sheath tumor
Source: PLoS One. 2017 May 30;12(5):e0178064. doi: 10.1371/journal.pone.0178064 (PMC5448771; doi:10.1371/journal.pone.0178064)
Supplement: S1 Table — The flow cytometer analysis with propidium iodide staining at 48 h after transfection of si-HIF-1α revealed a significant increase of subG1 fractions in MPNST cells under hypoxia. Experiments were performed in triplicate or more, and data are expressed as the mean ± SD. *P < 0.05. (DOCX) [file pone.0178064.s003.docx]

| **Cell lines** | **subG1 fraction**  **(Mean ± SD, %)** | **parent** | **si-control** | **si-HIF-1α** |
| --- | --- | --- | --- | --- |
| **FMS-1** | **Normoxia** | 2.08 ± 0.60 | 4.47 ± 4.71 | *9.85 ± 5.14 |
|  | **Hypoxia** | 4.87 ± 5.05 | 5.30 ± 5.35 | *13.22 ± 8.14 |
| **HS-Sch-2** | **Normoxia** | 2.28 ± 0.63 | 3.77 ± 2.52 | *9.17 ± 3.88 |
|  | **Hypoxia** | 4.33 ± 3.01 | 4.33 ± 3.15 | *12.5 ± 7.31 |
| **FU-SFT8611** | **Normoxia** | 1.22 ± 0.98 | 1.80 ± 0.79 | *4.00 ± 2.14 |
|  | **Hypoxia** | 2.67 ± 1.88 | 1.80 ± 0.85 | *8.00 ± 5.38 |
| **FU-SFT9817** | **Normoxia** | 2.60 ± 1.27 | 3.07 ± 2.27 | *4.75 ± 1.22 |
|  | **Hypoxia** | 2.97 ± 1.86 | 2.77 ± 1.74 | *8.08 ± 4.82 |

**S1 Table. Cell cycle assay with siRNA**

**P* <0.05
