# Supplementary material for: Hypoxia-inducible factor 1 alpha is a poor prognostic factor and potential therapeutic target in malignant peripheral nerve sheath tumor
Source: PLoS One. 2017 May 30;12(5):e0178064. doi: 10.1371/journal.pone.0178064 (PMC5448771; doi:10.1371/journal.pone.0178064)
Supplement: S2 Table — In the inhibitor kit provided by the Screening Committee of Anticancer Drugs (SCADS), eight compounds added at a concentration of 1 μM markedly inhibited the proliferation of more than one MPNST cell line compared with DMSO. In particular, chetomin, an inhibitor of HIF-1α, showed efficacy against three MPNST cell lines, and this result was consistent with the proliferation inhibitory effect exhibited by si-HIF-1α. (DOCX) [file pone.0178064.s004.docx]

| **Effectiveness** | **Compound names** | **Mechanism** |
| --- | --- | --- |
| **Four cell lines** | Staurosporine | Protein kinase C inhibitor |
| **Three cell lines** | ***Chetomin** | ***HIF-inhibitor** |
|  | Bortezomib | Proteasome inhibitor |
|  | YM155 | Survivin inhibitor |
| **Two cell lines** | Trichostatin A | Histone deacetylase inhibitor |
|  | Cucurbitacin | Janus activating kinase inhibitor |
|  | MG-132 | Proteasome inhibitor |
|  | Ouabain | Na/K/ATPase inhibitor |

**S2 Table. Inhibitor assay using SCADS Inhibitor Kit**
