# Supplementary material for: Hypoxia-inducible factor 1 alpha is a poor prognostic factor and potential therapeutic target in malignant peripheral nerve sheath tumor
Source: PLoS One. 2017 May 30;12(5):e0178064. doi: 10.1371/journal.pone.0178064 (PMC5448771; doi:10.1371/journal.pone.0178064)
Supplement: S3 Table — As the results of apoptosis analysis by flow cytometry using Annexin V-FITC and 7-AAD, chetomin significantly increased both apoptotic and necrotic fractions compared to DMSO in four MPNST cell lines. (DOCX) [file pone.0178064.s005.docx]

| **Cell lines** | **Fraction**  **(Mean ± SD, %)** | **Concentration of chetomin (nM)** | | |
| --- | --- | --- | --- | --- |
|  |  | **DMSO** | **50** | **200** |
| **FMS-1** | **Apoptosis** | 6.55 ± 3.13 | *17.88 ± 6.48 | *31.5 ± 14.36 |
|  | **Necrosis** | 8.88 ± 6.01 | 20.95 ± 12.98 | *42.13 ± 14.36 |
| **HS-Sch-2** | **Apoptosis** | 6.58 ± 6.30 | 13.68 ± 2.57 | *27.5 ± 7.72 |
|  | **Necrosis** | 4.4 ± 2.90 | 13.2 ± 8.01 | *44.97 ± 13.85 |
| **FU-SFT8611** | **Apoptosis** | 5.3 ± 2.43 | 7.6 ± 1.81 | *9.83 ± 5.33 |
|  | **Necrosis** | 1.9 ± 0.95 | 6.25 ± 6.86 | *12.23 ± 9.00 |
| **FU-SFT9817** | **Apoptosis** | 5.75 ± 1.40 | *14.55 ± 2.74 | *17.37 ± 3.97 |
|  | **Necrosis** | 5.05 ± 1.99 | *11.75 ± 2.54 | *21.93 ± 5.00 |

**S3 Table. Apoptosis induced by chetomin**

**P* <0.05
