# Supplementary material for: Hypoxia-inducible factor 1 alpha is a poor prognostic factor and potential therapeutic target in malignant peripheral nerve sheath tumor
Source: PLoS One. 2017 May 30;12(5):e0178064. doi: 10.1371/journal.pone.0178064 (PMC5448771; doi:10.1371/journal.pone.0178064)
Supplement: S4 Table — As the concentration of chetomin increased, cell cycle analysis with flow cytometer by propidium iodide staining revealed significant increase of subG1 fractions. These results suggested that chetomin induced cell death in the MPNST cell lines in a dose-dependent manner. (DOCX) [file pone.0178064.s006.docx]

**S4 Table. SubG1 fraction induced by chetomin**

| **Cell lines** | **Fraction**  **(Mean ± SD, %)** | **Concentration of chetomin (nM)** | | |
| --- | --- | --- | --- | --- |
|  |  | **DMSO** | **200** | **500** |
| **FMS-1** | **subG1** | 5.5 ± 6.42 | *27.67 ± 9.18 | *35.57 ± 14.70 |
| **HS-Sch-2** |  | 4.13 ± 2.41 | *21.67 ± 7.50 | *37.03 ± 5.13 |
| **FU-SFT8611** |  | 0.87 ± 0.35 | 0.73 ± 0.35 | *4.73 ± 3.70 |
| **FU-SFT9817** |  | 3.93 ± 2.89 | 8.47 ± 6.63 | *29.77 ± 6.77 |

**P* <0.05
